# Damage Detection in Flat Panels by Guided Waves Based Artificial Neural Network Trained through Finite Element Method

**DOI:** 10.3390/ma14247602

**Published:** 2021-12-10

**Authors:** Donato Perfetto, Alessandro De Luca, Marco Perfetto, Giuseppe Lamanna, Francesco Caputo

**Affiliations:** Department of Engineering, University of Campania “L. Vanvitelli”, Via Roma 29, 81031 Aversa, Italy; donato.perfetto@unicampania.it (D.P.); marco.perfetto1@studenti.unicampania.it (M.P.); giuseppe.lamanna@unicampania.it (G.L.); francesco.caputo@unicampania.it (F.C.)

**Keywords:** Artificial Neural Network (ANN), guided waves, Structural Health Monitoring (SHM), Finite Element Analysis (FEA), damage detection, metals, composites

## Abstract

Artificial Neural Networks (ANNs) have rapidly emerged as a promising tool to solve damage identification and localization problem, according to a Structural Health Monitoring approach. Finite Element (FE) Analysis can be extremely helpful, especially for reducing the laborious experimental campaign costs for the ANN development and training phases. The aim of the present work is to propose a guided wave-based ANN, developed through the use of the Finite Element Method, to determine the position of damages. The paper first addresses the development and assessment of the modeling technique. The FE model accuracy was proven through the comparison of the predicted results with experimental and analytical data. Then, the ANN was developed and trained on an aluminum plate and subsequently verified in a composite plate, as well as under different damage configurations. According to the results herein proposed, the ANN allowed to detect and localize damages with a high level of accuracy in all cases of study.

## 1. Introduction

Structural monitoring of primary and secondary structural elements is generally a complex, costly and time-consuming process. All structural components are inspected at regular intervals, using different non-destructive techniques (NDTs) that increase more and more the total duration of the downtime.

Such inspections for health-condition assessment are of the utmost importance for the safe and efficient operation of structures. A damage that cannot be reliably detected could not be repaired on time, leading to eventually catastrophic failures.

To overcome these problems, in view to ensure the safety of the structure, Structural Health Monitoring (SHM) systems able to monitor the actual structural integrity become necessary [1,2,3]. The use of a SHM system brings with it numerous benefits in terms of maintenance and repairing operations, including reduction in uncertainty in operators’ decision-making process, quasi-real time control of deficiencies and, thus, greater safety through continuous monitoring. Moreover, SHM will provide a dataset useful for maintenance phases, improving in the meantime the engineering capabilities and the future design of primary components with relative reduction in both medium- and long-term costs.

In the execution of SHM strategies, the most commonly used approach is that based on guided (or Lamb) waves (GW) [4,5,6] because of their powerful capability of long-distance propagation with high speed and little loss of energy. Several useful pieces of information can be derived from implementing a Lamb-wave-based identification associated with a damage detection method, such as qualitative indication of the occurrence of damage, quantitative assessment of the position of damage, quantitative estimation of the severity of damage and prediction of structural safety like the residual service life [7,8].

Successful damage identification by using a GW-based SHM system can be performed with transducers in a sparse configuration and using well-calibrated signal-interpretation techniques. Some essential steps are the following: (i) activating the desired diagnostic Lamb wave signal, using an appropriate transmitter and capturing the damage-scattered wave signals using a sensor or a sensors network in accordance with either the pitch-catch or the pulse-echo configuration; (ii) extracting and evaluating the characteristics of the captured wave signals with appropriate signal post-processing tools; (iii) establishing quantitative and/or qualitative connections between the extracted signal characteristics and the damage parameters (presence, location, geometric identity, severity, etc.); and (iv) figuring out the damage parameters of interest in terms of captured signals, based on the quantitative connections established in Step (iii) [8].

However, the main challenge of guided waves is that they propagate with multiple modes and exhibit a strongly dispersive behavior when the excitation frequency increases [9]. Thus, the attention is mainly paid on the zero-order symmetric and antisymmetric modes, $S_{0}$ and $A_{0}$, respectively. Both the $S_{0}$ and $A_{0}$ modes are sensitive to structural damage, and both can be used for identifying damage, though the $S_{0}$ mode exhibits higher sensitivity to damage in the structural thickness, fatigue damage and delamination. Meanwhile, the interpretation of the signals can be very challenging, limiting the extraction of useful information on damages. Thus, a large number of experiments is required to properly identify GW characteristics. To reduce time and costs, chirp broadband signals can be used, together with the Finite Element Method (FEM), semi-analytical tools, etc.

With the latest advances in neurosciences and high-capability computing devices, machine learning (ML) algorithms based on Artificial Neural Networks (ANNs) have rapidly emerged as a promising tool to solve damage/defects identification and localization problem [8,10]. Various techniques, including mode shapes, natural frequencies, strain history and many others [11,12,13,14,15], have been used for damage identification during the years for different application fields. This was achieved by utilizing different supervised or unsupervised machine learning techniques for damage recognition [16,17,18].

Recent research has focused on damage quantitative estimation by using Lamb wave signals features in combination with ANN and FEM in order to ensure the accuracy of ANN (strictly related to the data used to train the network) [10,16]. A Lamb-wave-based damage evaluation method assisted by an ANN model was presented by Qian et al. [19], using Damage Indexes related to changes in amplitude and phase of recorded signals. They found a good agreement between experimental and predicted results in a carbon fiber-reinforced polymer composite plate. Sharif-Khodaei et al. [20,21] developed an Artificial Neural Network (ANN) that is able to locate impact of different energies in a complex structure, such as a composite-stiffened panel. Such an ANN was trained by means of a large number of impact simulations, based on FEM, covering a wide range of impact energies and locations. De Fenza et al. [10] used GW propagation and ANN to determine the location and the degree of damage in a metallic plate: it was divided into sectors, and for each one, the probability of occurrence of the damage was assessed.

Obviously, the performance of an ANN strictly depends on the size and complexity of the obtained dataset. A very large or high-dimensional dataset could affect the accuracy of the classification or clustering and make the model computationally expensive. Employing data-preprocessing methods when dealing with high-dimensional sensory data is also of great importance and impacts the ANN performance [18].

In this paper, a machine learning approach based on ANN is proposed in order to demonstrate and discuss the usability of artificial intelligence for the purpose of detecting and localizing a damage and its coordinates. A physical experiment and numerical simulations on an aluminum plate were carried out. The aluminum plate was equipped with four piezoelectric transducers to activate and receive the wave response in baseline (no damage) and actual (damaged) configurations of the structure.

As the first step, the development and assessment of the Finite Element modeling technique was addressed. The FE model accuracy was proven through the comparison of the predicted results with experimental and analytical data in terms of $S_{0}$ and $A_{0}$ group velocities’ curves.

Once established with respect to GW propagation, the numerical data were used for the training and validation process of the ANN. In particular, starting from the $S_{0}$ wave packet extracted from the numerical dataset through a post-processing technique, Damage Indexes (DIs), under different damaged configurations (in terms of both position and dimension), were calculated. High value of DI means a damage very close to the corresponding actuator–receiver path [22]. This was possible through the development of an in-house Matlab^®^ (The MathWorks Inc., Natick, MA, USA) code that automatically extracts the $S_{0}$ mode from the baseline and actual states of the structure and calculates the DIs. The DIs dataset was used as input for the ANN, while the targets are represented by the coordinates of the centers of the modeled damages. Achieved results prove that the ANN can predict the damage location precisely.

Then, the developed ANN was verified on a CFRP (carbon fiber-reinforced polymer) plate. According to the results herein proposed, the ANN allowed to detect and localize damages with a high level of accuracy in all cases of study.

The trained algorithm could be directly applied to experimental measurements without the need of retraining.

The layout of the paper is as follows. Details of the case study are outlined in Section 2. Section 3 deals with the numerical methodology, based on FE, for a proper modeling of the wave propagation mechanisms; dispersion curves (experimental, numerical and semi-analytical) of the zero-order modes are shown and compared. The description of the ANN-based damage detection procedure, using Damage Indexes, is detailed in Section 4. Finally, the performance of the ANN and achieved results are discussed in Section 5 for an aluminum and a composite panel under several damaged configurations. Section 6 concludes the paper.

## 2. Case Study

GW propagation mechanisms were investigated in a simple flat isotropic panel. For isotropic materials, Lamb waves travel with the same velocity omni-directionally, and the wavefronts form a circle. This case study, although simple, represents a starting point for the development of an ANN useful for damage detection.

**Figure 1:**
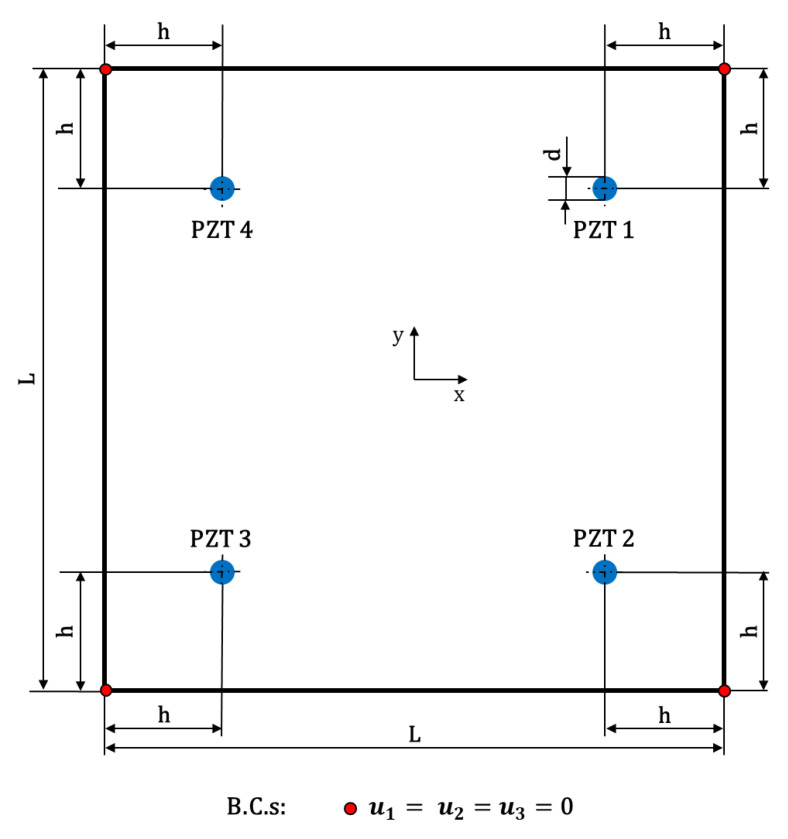
Schematic of the geometry and map of PZTs network.

The plate under investigation has a square shape with dimension L=287 mm, and a thickness of $t_{a}=2\;\mathrm{mm}$ (Figure 1). The mechanical properties are listed in Table 1. A four Circular DuraAct (PI Ceramics) PIC255 piezoelectric transducers network was used for both the actuation and sensing of Lamb waves. The thickness and the radius of the PZT wafers are $t_{\mathrm{PZT}}=0.5\;\mathrm{mm}\;$and $d_{\mathrm{PZT}}=10\;\mathrm{mm}$, respectively. The PZTs, whose mechanical properties are listed in Table 1, were surface-mounted onto the specimen. In particular, they are located at a distance h=55 mm from the edges (Figure 1). The adhesive EA 9466 from Loctite (Henkel AG & Co. KGaA, Düsseldorf, Germany) was used to bond the transducers.

In order to reduce the number of experiments to carry out, a chirp signal was used. The chirp signal is given as follows:

(1) $$V_{\mathrm{chirp}}\left(t\right)=V_{\mathrm{in}}\left[H\left(t\right)-H\left(t-t_{\mathrm{chirp}}\right)\right]\sin\left(2\pi \left(f_{0}t+\frac{f_{1}-f_{0}}{t_{\mathrm{chirp}}}t^{2}\right)\right),$$

where $t_{\mathrm{chirp}}=0.25\;\mathrm{ms}$ is the duration of the chirp signal, $f_{0}\;$= 50 kHz is the start frequency, $f_{1}\;$= 500 kHz is the end frequency, $V_{\mathrm{in}}$ is the input amplitude and H is the Heaviside function. The chirp signal allows users to achieve in a single test all dispersion curves in the selected frequency band. The tone-burst response, preferred due to the dispersive nature of Lamb waves [23], was then extracted by using the reconstruction procedure described in Reference [24] to allow for the comparison for each frequency.

A 16 V peak-to-peak input amplitude was applied to the actuator PZT, using a TiePie waveform generator, and the TiePie Digital Oscilloscope was used to record the signals acquired at the sensor PZTs with a sampling frequency of 2 MHz. The total recording duration of the experimental signals is $\mathrm{tot}=2\times 10^{-4}\;s$, and each measurement is recorded 32 times and averaged to improve the signal to noise ratio. The acquired signals from all four channels have a resolution of 12 bit. Each measurement was 0.200 ms long.

## 3. Numerical Approach for Wave Propagation Modeling

In this section, at first, FE modeling techniques are compared in order to prove the efficiency of 2D-Shell elements [25,26] in simulating GW propagation in the isotropic plate under different frequencies against 3D ones. In fact, especially for complex structures, the modeling via 3D-Solid Finite Elements can be prohibitive in terms of computational costs. Thus, there is a need to adopt, as far as possible, shell elements.

Once established against experiment or analytical data, the FE model can be used to predict wave-propagation mechanisms in a damaged configuration of the plate, and to get a useful dataset for the training of the ANN.

In this work, numerical data were compared to the experimental ones, using sensor 1 as actuator and the others as receivers. Such a comparison, although simple and well-studied, offers the opportunity to validate the numerical models and evaluate their convergence by using an isotropic material.

The geometry of the panel under investigation, described in Section 2, was modeled in Abaqus^®^ CAE environment (Dassault Systems Simulia Corp., Providence, RI, USA). Three different element types from the Abaqus^®^ Finite Elements library were considered: (i) 2D-Shell (conventional shell), S4R element with 6 degrees of freedom per node; (ii) 3D-Shell (continuum shell), SC8R element with 3 degrees of freedom per node; and (iii) 3D-Solid (brick element), C3D8R element with 3 degrees of freedom per node.

In order to reduce the number of simulations, a chirp excitation signal was modeled in the frequency range (50÷500 kHz), as described in the previous section. Moreover, it is well-known from the literature that, to correctly characterize the scattering phenomena of Lamb waves across a modeled damaged area, a minimum number of 8–10 nodes per wavelength (NPW) should be set [8]. Thus, for the plate and sensor average element size evaluation ($l_{e}^{\mathrm{plate}}$ and $l_{e}^{\mathrm{pzt}}$, respectively), two different values of NPW under 500 kHz carrier frequency (it is the actual end frequency of the chirp signal) were considered: 10 NPW, corresponding to $l_{e}^{\mathrm{plate}}=0.60\;\mathrm{mm}$, $l_{e}^{\mathrm{pzt}}=0.37\;\mathrm{mm}\;$and step size $t_{\mathrm{inc}}\left(10\;\mathrm{NPW}\right)=1\times 10^{-7}\;s;$ and 20 NPW, corresponding to $l_{e}^{\mathrm{plate}}=0.30\;\mathrm{mm}$, $l_{e}^{\mathrm{pzt}}=0.18\;\mathrm{mm}$ and step size $t_{\mathrm{inc}}\left(20\;\mathrm{NPW}\right)=5\times 10^{-8}\;s$.

This led to the development of different FE models: two 2D-Shell based, providing 10 and 20 NPW respectively; two 3D-Shell based, providing 10 and 20 NPW respectively; and only one 3D-Solid based, providing 10 NPW (to reduce the computational costs). In all the numerical analyses, the chirp actuation signal was imposed. This means that, for example, taking into account $l_{e}^{\mathrm{plate}}=0.60\;\mathrm{mm}$, the FE analysis discretises 100 NPW under 50 kHz reconstruction frequency. For the sake of simplicity, the FE models are just named 10 and 20 NPW, respectively.

The wave propagation is a dynamic phenomenon, so the explicit environment was chosen to simulate the actuation, propagation and sensing of GW. For this reason, to ensure the accuracy of the numerical solution, the time step must be less than the ratio of the minimum distance of any two adjacent nodes to the maximum wave velocity (often the group velocity of the ${\;S}_{0}$ mode), as follows:

(2) $$t_{\mathrm{inc}}=\frac{l_{e}^{\min}}{{\;C}_{g}},$$

where $l_{e}^{\min}$ is the size of the smallest element, and $C_{g}$ is the group velocity [27].

**Figure 2:**
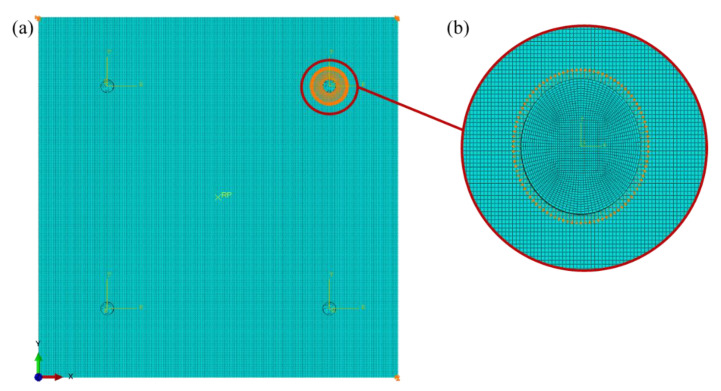
(**a**) FE model of the aluminum plate under study in Abaqus^®^ CAE. (**b**) Focus on sensor mesh and GW input signal.

The FE model is reported in Figure 2. For the actuation and receiving signal, the sensors network was numerically modeled as in the experiment in terms of location and material/geometry configurations. Each sensor was modeled by means of reduced integration solid elements (C3D8R), while 3 elements were modeled through the thickness. To ensure the contact between sensors and plate, a node-to-surface contact formulation was employed at the “tied” interfaces to simulate the adhesive layer between sensors and plate (not here modeled) [25].

Finally, the translational degrees of freedom of the 4 corners of the plate were constrained as in the experiment, while, relative to the GW propagation, radial displacements equivalent to the input voltage of Equation (1) were calculated through Equation (3):

(3) $$d_{r}=Q_{V}\;V,$$

where $Q_{V}$ is a conversion constant for the actuation, depending on the plate and sensor geometry and material properties, as widely described in Reference [8].

This effective displacement was applied on the upper actuator edge (see also Figure 2b) after having defined a proper polar coordinate system at the center of the actuator, as effectively reported in Reference [8].

The implicit scheme could be used to handle either the wave propagation or mechanical–piezoelectric problems. In fact, there is the option of using electric coupling PZT Finite Elements. That means that the voltage can be directly applied to the terminals of the transducers, and the corresponding strain response in the sensors is acquired through the electro-mechanical coupling of the elements (Equation (3)). Such elements, however, are not available for the explicit procedure used herein to perform the analyses. A comprehensive overview about the modeling of PZT can be found in References [8,23,28,29,30,31].

### Dispersion Curves

The wave propagation was simulated in the Abaqus^®^ explicit environment. The test case consists of a multi-frequency analysis with a chirp actuation signal, activated by ‘PZT 1’, due to isotropy of the plate, according to Equation (1). To avoid mode superimposition, low-frequency Lamb waves should usually be chosen for damage detection. In this study, the excited signal is a chirp one limited to the frequency range 50÷500 kHz. Then, in order to concentrate the majority of the wave energy on a specific central frequency, recorded data were reconstructed by means of a n-cycles sinusoidal tone-burst Hanning windowed signal with a step of 25 kHz (50:25:500 kHz), allowing reducing spectrum leakage.

Numerical results in the selected frequency range were compared to the experimental ones and also to those obtained by means of the Dispersion Calculator (Center of Lightweight Production Technology, German Aerospace Center (DLR), Augsburg, Germany) [32]. It is a Matlab^®^-based general-purpose tool that interactively allows users to create dispersion curves that simply define the material model and properties. It computes the phase and group velocity dispersion, as well as internal stress and displacement fields (mode shape) of Lamb and shear horizontal waves in isotropic and multilayered composites.

**Figure 3:**
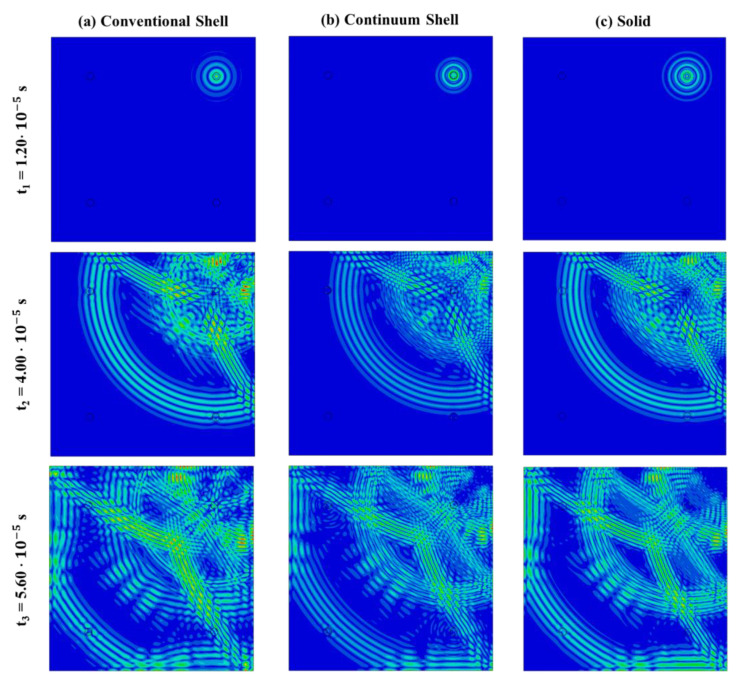
Guided wave propagation at 3 time instants in the aluminum plate modeled by the three different element types—300 kHz carrier.

Displacements field contour plots in Figure 3 show the wave propagation under 300 kHz carrier test for the three investigated FE models. As expected for an isotropic material, the propagation speed is equal along all material directions and the guided waves propagate in a circular pattern. Differences between 2D-Shell, 3D-Shell and 3D-Solid can be attributed to the different representation of the panel boundaries, as also noted in References [33,34].

Predicted signals, recorded at sensor locations, were calculated as the average of the in-plane strains, $\;\bar{\varepsilon }\;$, reads by all nodes defining each sensor. Similar to Equation (3), accordingly to piezoelectric relations, the voltage in PZTs can be calculated through the strain measurements as follows:

(4) $$V=Q_{s}\;\bar{\varepsilon },$$

where $Q_{s}$ is a conversion constant for the sensing (further mathematical details can be found in Reference [8]). Then, converted signals were processed by means of the developed code and compared in terms of ToF and amplitudes.

**Figure 4:**
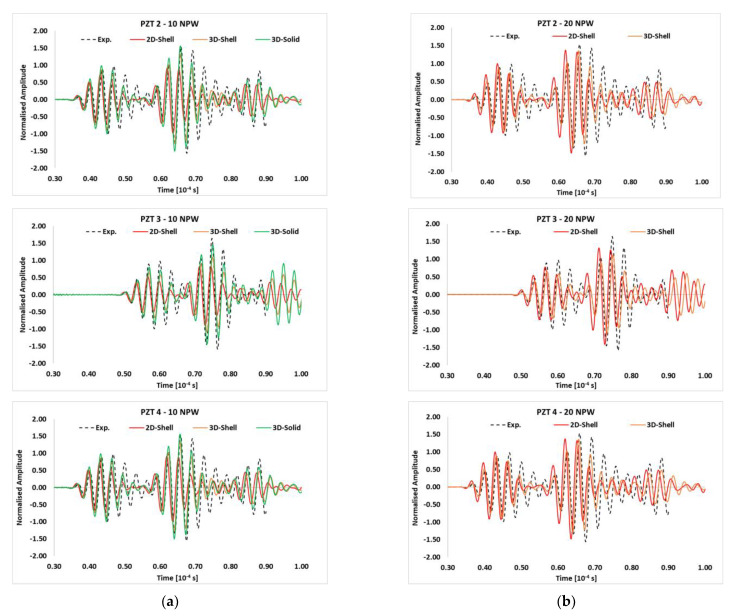
Comparison of the received signals at each PZT location for the conventional shell, continuum shell and solid FE models with the experimental data adopting (**a**) 10 NPW and (**b**) 20 NPW—300 kHz carrier.

To validate the Finite Element models, the responses of the transducers were compared for the numerical and experimental results. In particular, for the sake of brevity, in Figure 4, only signals reconstructed under the 300 kHz carrier were compared to the respective experimental ones. The same level of accuracy was achieved for all frequencies. Due to the material symmetry, signals recorded at PZT 2 and PZT 4 are the same. In all three cases, the numerical predictions are able to approximate the arrival of the first wave packet with adequate accuracy for both 10 (Figure 4a) and 20 (Figure 4b) NPW. According to Figure 4, each signal was normalized with respect to the maximum amplitude of its own $S_{0}$ wave packet. This allows highlighting the differences between predicted and experimental data.

It is noted that attenuation phenomena were not considered in this study. Additionally, the adhesive layer between the PZT wafer and the panel was modeled as a rigid link. These effects can influence the amplitude of the received waves and justify the slight mismatch existing between measured and predicted data [33,34].

Once the distances between actuator/receivers and the ToF on all paths are known, it is possible to calculate the velocity of Lamb waves packet (𝑐_𝑔_) [35]. Considering that for an isotropic material, 𝑐_𝑔_ does not depend on propagation’s direction, to compute the group velocity, only the path actuator 1-receiver 2 was considered. As highlighted in Table 2, all three element types can make group velocity estimations with small relative error compared to the experimental measurement. As expected, the 3D-Solid modeling procedure permit to achieve more accurate results, but the error associated with the 2D-Shell elements is relatively small and can be considered widely acceptable.

To assess the proposed numerical procedure, the extracted dispersion curves were validated against the experimental one and those provided by Dispersion Calculator Software. Effectively, curves extracted from Dispersion Calculator were defined through a semi-analytical method.

**Figure 5:**
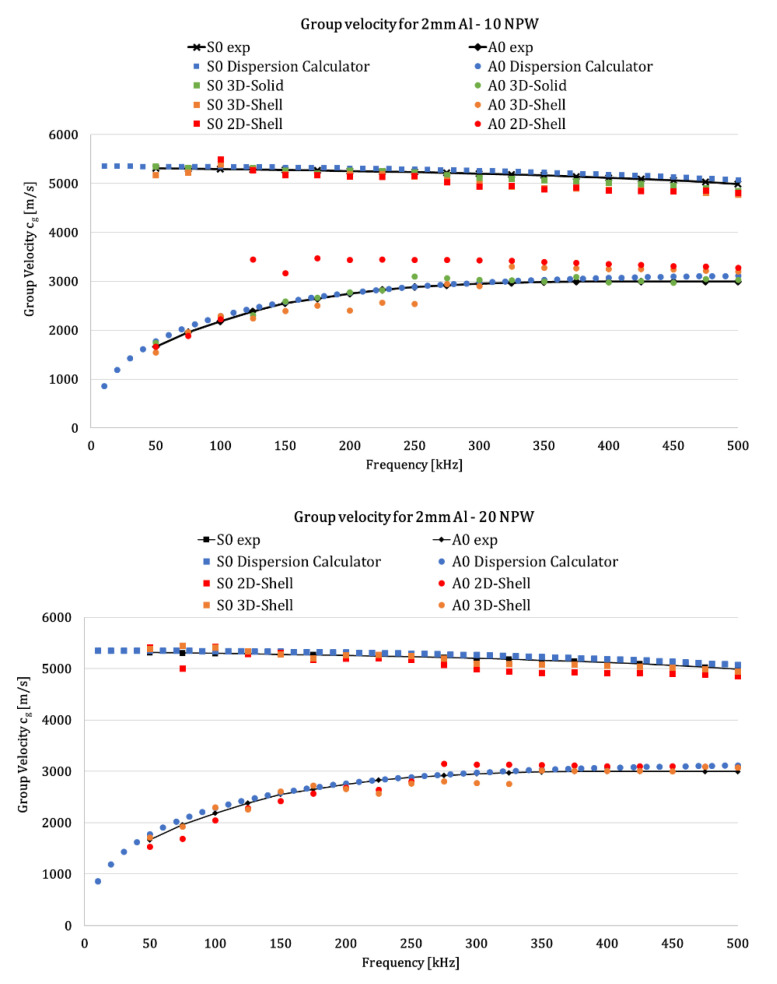
S_0_ and A_0_ modes’ dispersion curves in the flat aluminum panel for 10 and 20 NPW.

**Figure 6:**
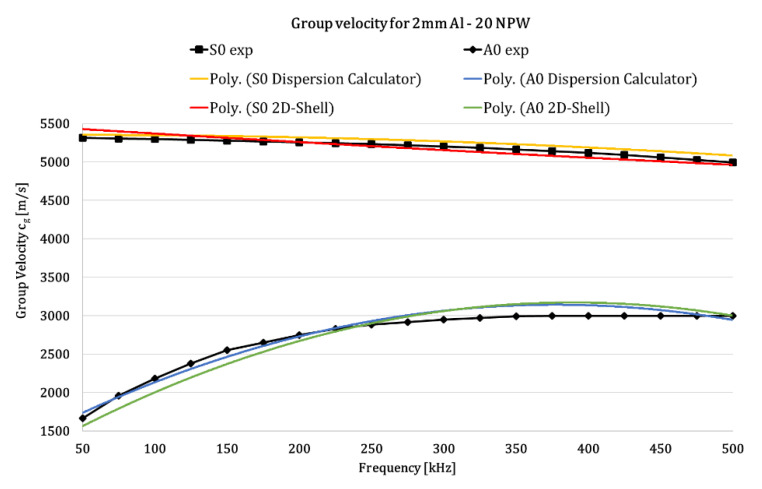
Dispersion curves for the S_0_ and A_0_ modes—2nd-order polynomial fitting curves—20 NPW.

**Figure 7:**
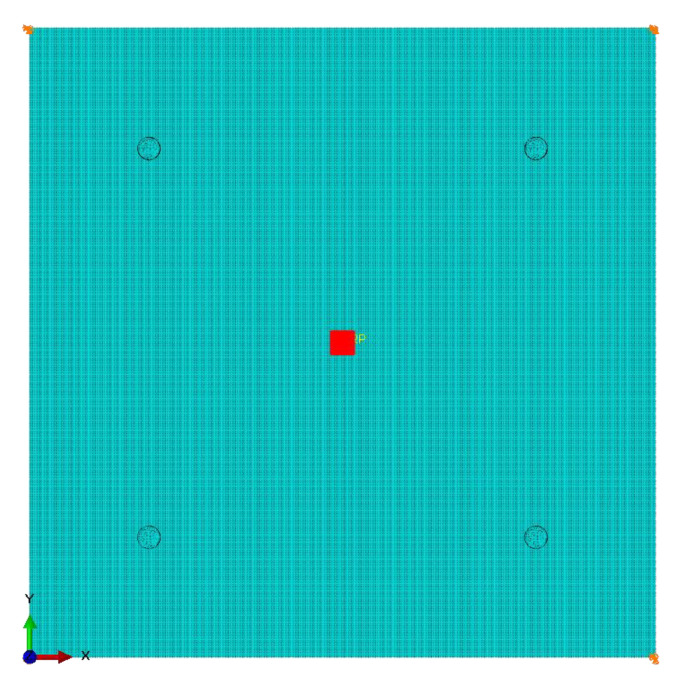
Example of FE damage modeling: degradation of the elastic properties (−70%) of the elements set highlighted in red.

**Figure 8:**
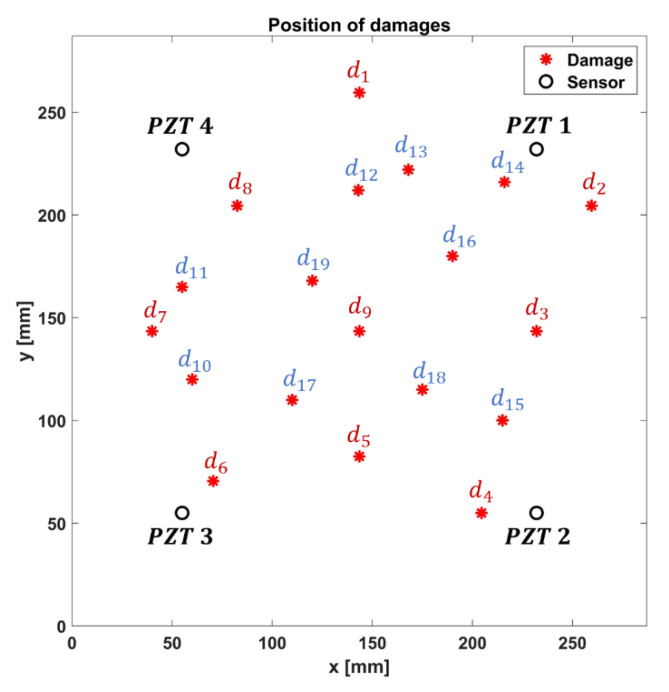
Sensors (black circles) and damages (red stars) map.

**Figure 9:**
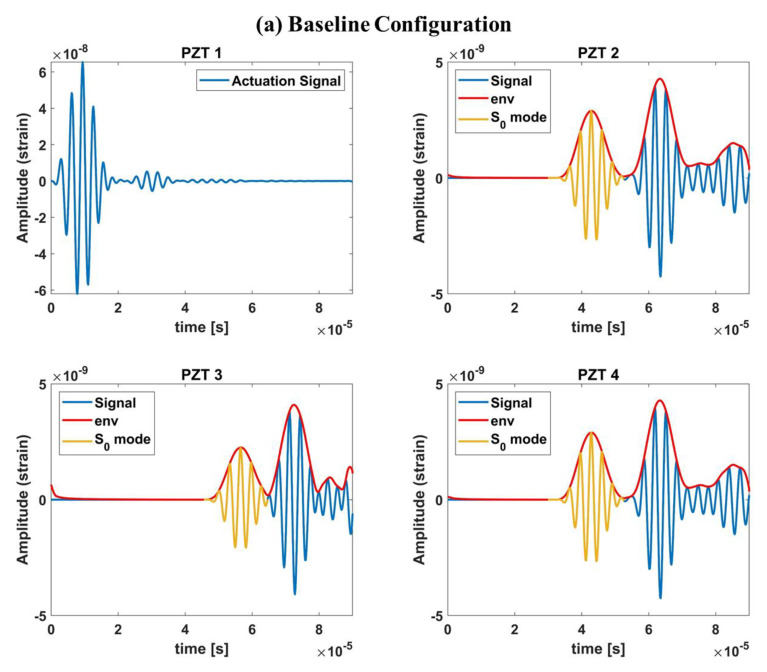

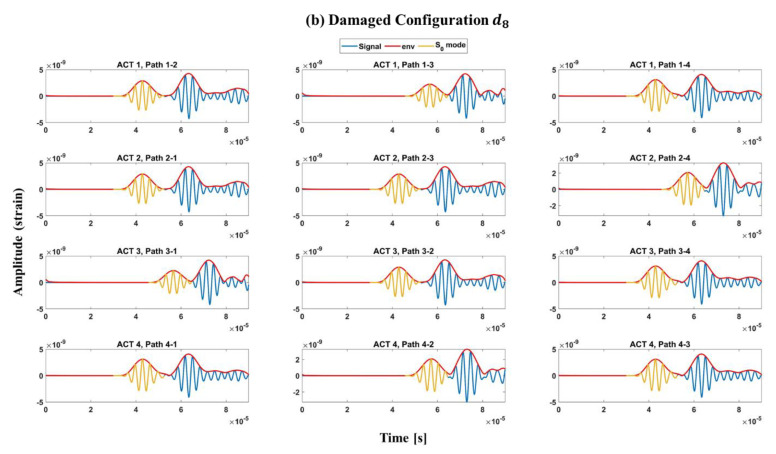
S_0_ mode extraction from baseline and actual configurations. Example for damage in position d_8_.

**Figure 10:**
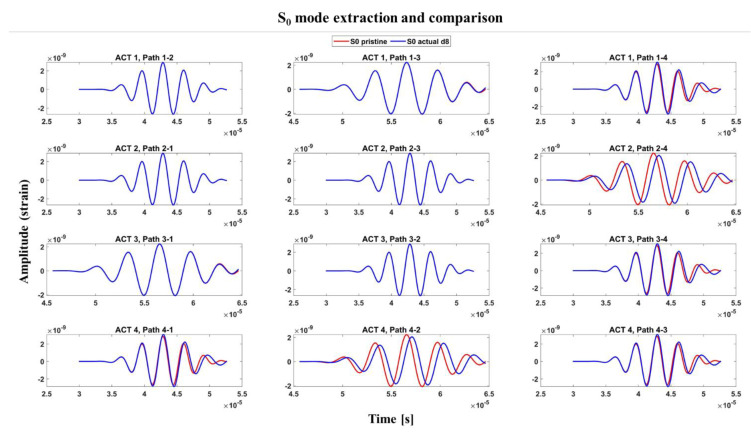
Comparison between the S_0_ modes for the baseline and the actual configurations. Example for damage in position d_8_.

Guided wave dispersion curves for the $S_{0}$ and $A_{0}$ modes are reported in Figure 5, in which dots represent the extracted values. As visible from Figure 5—10 NPW—the conventional shell FE model (red dots) is not capable of accurately predicting the group velocity of the $A_{0}$ mode. Moving to 20 NPW, a good agreement between experimental, semi-analytical and numerical data was found for all of the developed FE models, demonstrating the good modeling of the wave-propagation phenomenon.

Because of the good level of agreement, in order to reduce the computational costs with respect to the 3D modeling technique, the 20 NPW 2D-Shell FE model can be adopted to easily and accurately extract the dispersion curves for both the $S_{0}$ and the $A_{0}$ modes in an isotropic panel, as is visible from Figure 5. 

Focusing on the 20 NPW 2D-Shell FE model and its comparison to the experimental and semi-analytical data, it can be observed that, by interpolating these points with an 2nd-order polynomial curve, the trend of the dispersion curve can be obtained. As it can be seen from Figure 6, the numerical analyses provide very accurate results: the trend of the polynomial curves (solid lines) interpolating numerical data (dots of Figure 5) matches very well the 2nd-order polynomial related to experiments and semi-analytical values, and this is true for both $S_{0}$ and $A_{0}$ modes. Even if the $A_{0}$ mode is slower than $S_{0}$, due to reflections being more difficult to detect, the numerical trend is very similar to the actual one, confirming the capability of the algorithm.

Lastly, considering the 2nd-order polynomial interpolations, the percentage differences with respect to the experimental and semi-analytical values were computed (Table 3 and Table 4, respectively). For the $S_{0}$ mode, the absolute percentage difference does not exceed the 3%, while, for the $A_{0}\;$mode, it is limited to 12%, demonstrating, again, the efficiency of the numerical modeling and the post-processing procedure.

## 4. ANN-Based Damage Detection Procedure

### 4.1. Damage Indexes

Once we established the capability of the shell elements in simulating GW propagation behavior, square-shaped damages were introduced by degrading the elastic material properties of the corresponding Finite Elements (softening technique) [36] of about 70% (Figure 7). This technique allowed achieving a good agreement with reference to the experiments, as well as reducing the modeling efforts with reference to the deleting technique [36].

A wide numerical campaign was set up in order to get a useful dataset for the training of the ANN. In particular, the dataset consists of the signals reconstructed under a central frequency 300 kHz. The chosen frequency enables the localization of damage with a diameter greater than 7 mm more accurately (in recognition of the fact that the half wavelength of a selected wave mode must be shorter than or equal to the damage size to allow the wave to interact with the damage) [37,38]. On the other hand, as the frequency decreases, the wavelength increases, making the minimum size of localized damage higher and higher.

Thus, square-shaped damages with a size of 5 and 10 mm were modeled at different points of the plate, as better specified in Table 5. The coordinates are expressed by considering the bottom left-hand corner of the plate as the origin of the Cartesian coordinates system, Figure 7. Figure 8 shows the positions of the damages (Table 5) on the plate (those common to both damage dimensions). As visible, some damages external to the area covered by the sensors network were also considered for the definition of the training set in order to improve ANN efficiency.

The signals were collected in a round-robin manner, where one transducer acts as actuator and the others act as sensors until the signals from all the receivers are collected. The presence of a damage alters the wave propagation, causing clear changes in the damaged wave packets with respect to the healthy ones. In particular, the change in amplitude and time of flight can be used to quantitatively indicate the most affected path [22].

Thus, the first step in the damage-detection phase is to extract the $S_{0}$ mode in the pristine configuration and then in the “faulty” configuration. This was achieved by implementing an in-house code that allowed us to extract the $S_{0}$ mode automatically without the need for visual graphical support. For the sake of brevity, an example is reported in Figure 9. The blue lines represent the recorded signals, the red one is the envelope and the yellow-colored part of the signal is the extracted $S_{0}$ mode.

Figure 10 shows a comparison between the $S_{0}$ mode extracted from the two configurations, pristine and damaged, in which it is possible to notice how the amplitude of the signal recorded in the actual configuration changes. Such a comparison is quantified through the evaluation of a Damage Index, DI. Specifically, in this work, the DI given by Equation (5) was used [22]:

(5) $$\mathrm{DI}=\sqrt{\sum_{i}\frac{\left(C_{p}^{2}-C_{d}^{2}\right)}{C_{p}^{2}}},$$

where $C_{p}$ and $C_{d}$ are respectively the amplitude of the signal, at same time, in the configuration “pristine” and “damaged”. The high value of the Damage Index means that the damage is placed along or close to the corresponding actuator–sensor path. Conversely, low Damage Index means that the damage is far from the actuator–sensor path.

**Figure 11:**
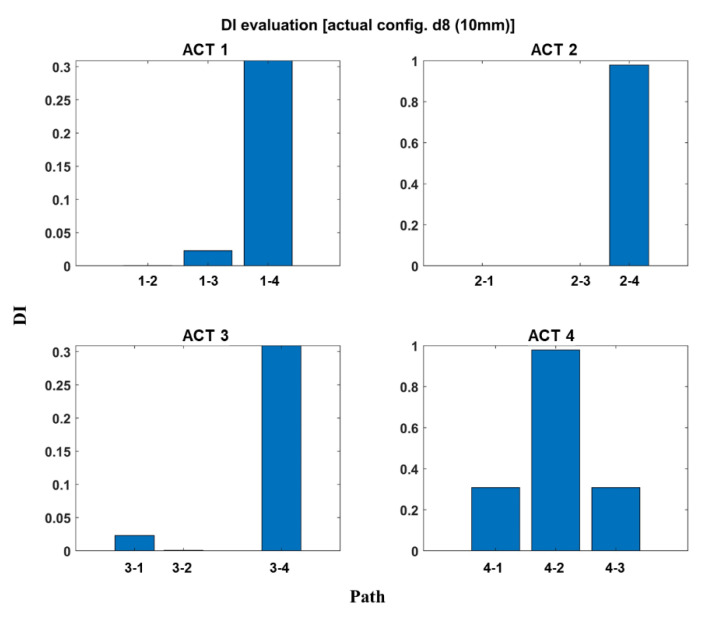
DI evaluation (damage of 10 mm). Example for damage in position d_8_.

The developed code also allows automatically calculating the Damage Index for each actuator–sensor path. The Damage Indexes obtained for each damage condition were normalized with respect to the maximum value among all paths for the different actuators. An example is reported in Figure 11 for the damage in position $d_{8}$. As visible from the figure, the DI enables the identification of the most affected paths. Moreover, by knowing the damage position, the DIs suggest that the damage is localized closer to the PZT 4, since, when this sensor is used as an actuator, the values of DI are much higher.

### 4.2. ANN Modeling

An Artificial Neural Network requires datasets for building up the input-output relation. In particular, as previously described, through an in-house code, it was possible to extract signal features for the input vector $x_{j}$. The ANN approach used in this work is based on the Damage Index of Equation (5) [22] that was described in the previous section. Therefore, the N-dimensional input vector consists of the DIs related to each sensing (actuator–receiver) path. The output vector instead includes the (x, y) coordinates of the localized damage. After generating the input and output vectors, the architecture of the ANN can be defined. It is clearly stated that the efficiency of the ANN increases when the dimension of the dataset increases. However, at the same time, this leads to an increase of the computational cost.

**Figure 12:**
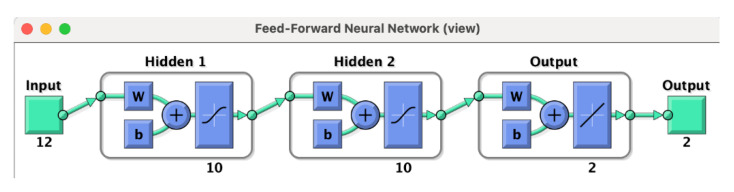
Architecture of the ANN includes one input layer, two hidden layers and one output layer; w, weights vector; b, bias vector (the image was produced by the MATLAB’s ANN toolbox).

As shown in Figure 12, the feed-forward neural network herein developed contains one input layer of 12 features (4 sensors and 3 paths, for a total of 12 inputs for each damaged configuration) and one output layer of two features, namely the damage center coordinates (x, y). Two hidden layers with multiple (10) neurons were defined to connect the input and output data, resulting in a fully connected neural network, according to the literature [39]. Each k-th neuron of the hidden layer is connected with all neurons of the previous layer, and its output $z_{k}$ can be described as follows:

(6) $$z_{k}=\varphi \left(\sum_{j=0}^{N}W_{\mathrm{kj}}x_{j}+b_{k}\right),$$

where $x_{j}$ is the value of the input vector at the discrete position, with j =1,2,…N; $W_{\mathrm{kj}}$ is the weight connected to each neuron k at the discrete position, with j =1,2,…N; φ is the transfer (activation) function; and $b_{k}$ is the bias value.

## 5. ANN Results and Discussion

### 5.1. Training of the ANN

In the current work, the Levenberg–Marquardt algorithm was used to train the ANN, as it appears to be the fastest method for training moderate-sized feedforward neural networks [40]. This algorithm is one of the variations of the back propagation algorithm, which is a gradient descent method in which the network weights are moved along the negative of the gradient of the performance function. In the current case, the chosen performance function is the mean squared error (MSE). The learning problem is considered to be solved when the combination of weights is able to minimize the error function.

**Figure 13:**
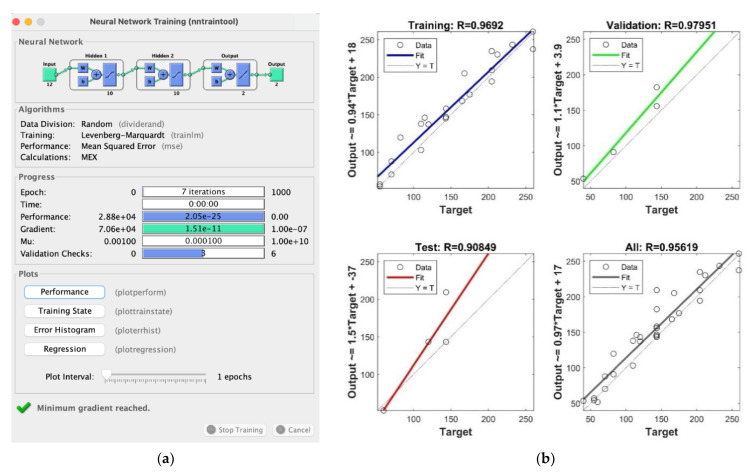
(**a**) ANN performance and **(b**) regression plot.

The results of this neural network (Figure 13a) show that the gradient is close to zero, as expected, as well as the performance parameter. The µ (Mu) parameter is the algorithm damping factor: it decreases after each successful step (reduction in the performance function), while it increases when a tentative step would result in a performance function increment. In this case, it starts from 0.001 as initial default value, and it decreases with a factor of 0.1, ending with a value of 0.0001.

The neural network was trained with fifteen of the nineteen 10 mm damage conditions, while the remaining four were used for validation and testing purposes. Figure 13 shows, as well, the performance obtained during the training, considering that the training was carried out in several steps on different datasets (always 15 of 19) in order to avoid the overspecialization of the network.

The ANN validation step consisted of the evaluation of the regression plot, which simply shows the relationship between the outputs of the network and the defined targets [41]. In detail, if the training were perfect, the network outputs and the targets would be exactly equal, but the relationship is rarely perfect in practice, especially when a limited dataset is adopted. Three regression plots for training, validation and testing data, together with the one for the overall dataset, are reported in Figure 13b. The dashed line in each plot represents the perfect result, i.e., outputs = targets. The solid line represents the best fit linear regression line between outputs and targets. The R value is an indication of the relationship between the outputs and targets. R=1 indicates that there is an exact linear relationship between outputs and targets, while if R is close to zero, then there is no linear relationship between outputs and targets. For the network herein proposed, the data indicate a good fit: the network is sufficiently accurate even though a limited dataset was used for the training phase.

### 5.2. ANN Validation and Tests

**Figure 14:**
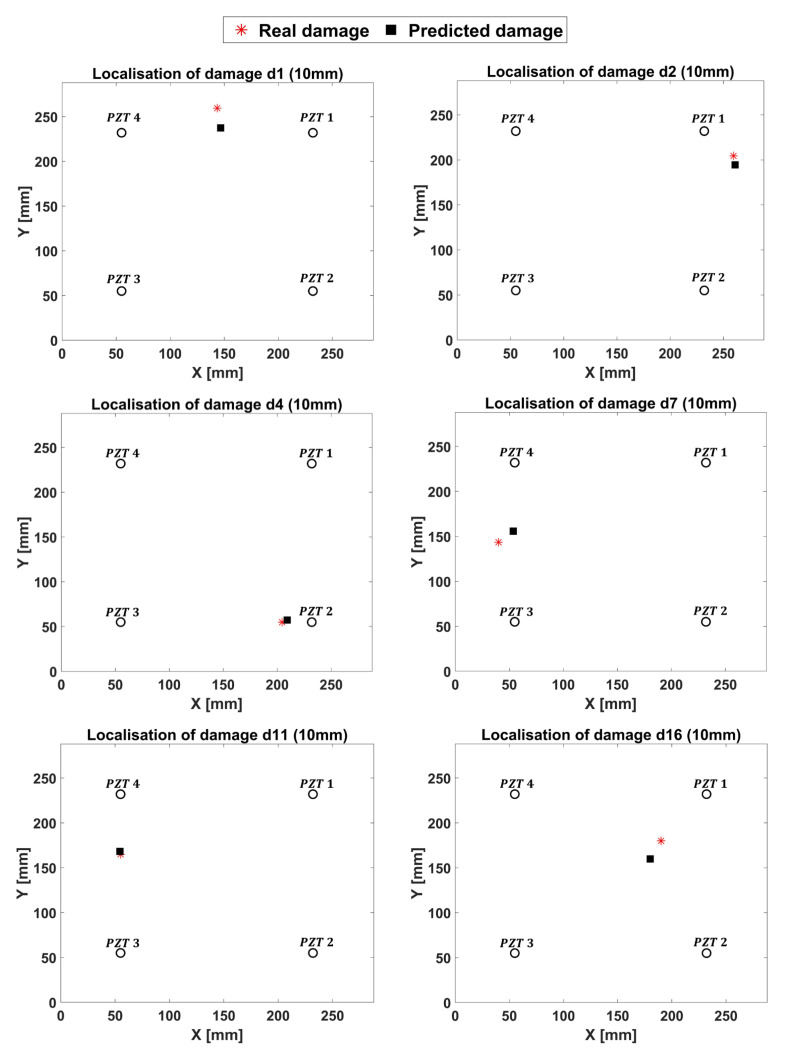
Results of ANN (10 mm damage).

**Figure 15:**
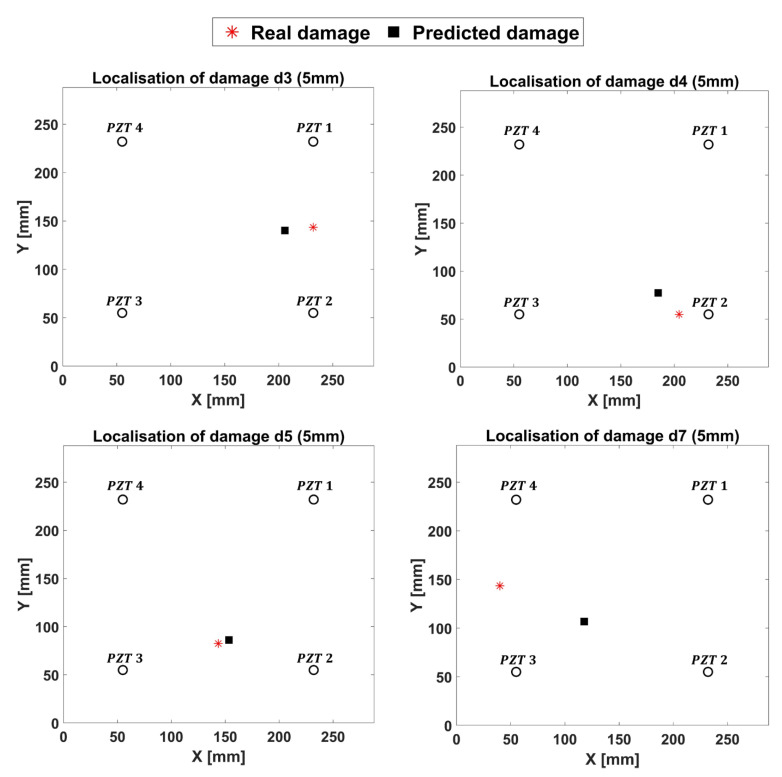
Results of ANN for the 5 mm damaged configurations.

In this section, the results provided by ANN are shown. Specifically, the fully trained ANN was validated by means of the remaining four 10 mm damaged test cases. Figure 14 shows a good agreement between the predicted damage locations and the modeled ones. When the inputs do not belong to the training set or to a neighborhood of it, the networks provide results that are different from the expected ones but comparable to them (Figure 14). Thus, the 5 mm damage dataset, not adopted for the training procedure, was used to test the ANN. It is herein underlined that, under 300 kHz carrier frequency, the minimum detectable damage should be 7 mm. Thus, the capability of the ANN to identify and successfully localize damages smaller than the defined threshold must be verified. According to Figure 15, the ANN is also capable of predicting smaller damages; however, it does so with a reduced but acceptable accuracy.

### 5.3. ANN for a Damaged Composite Panel

The ANN developed, tested and used for damage detection for the aluminum panel was herein used also to predict the damage location in a CFRP (carbon fiber-reinforced polymer) composite panel.

The CFRP composite panel is made of eight layers with stacking sequence [0/90/+45/−45]_s_. The thickness of the plate is t=1.5 mm, while the horizontal and vertical dimensions are the same of the aluminum panel of Figure 1. The 0° fiber direction is oriented along the 𝑥-axis. Each layer of the prepreg is assumed to behave as an orthotropic material, and the mechanical properties of the lamina are listed in Table 6.

**Figure 16:**
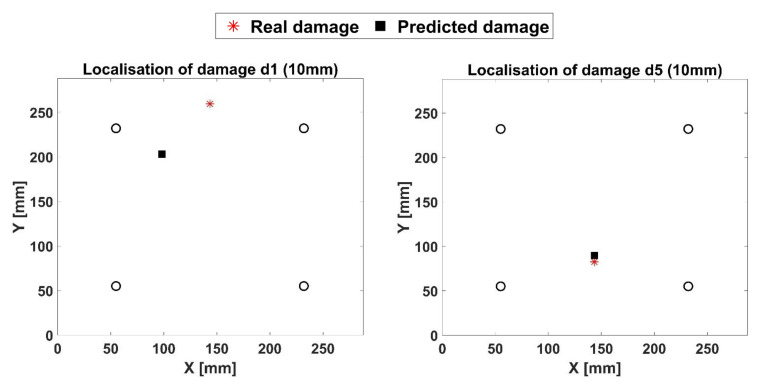

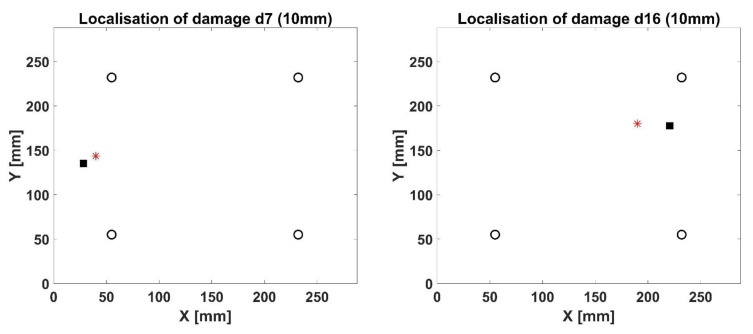
Results of ANN for the composite 10 mm damaged configurations.

In detail, the 20 NPW 2D-Shell FE modeling approach was chosen for the plate, while the 10 mm damage was modeled again by degrading the elastic material properties of the corresponding Finite Elements (softening technique) of about 70%. In this case, four damaged configurations, as shown in Figure 16, were investigated separately. The ANN was applied to all of these new cases without any further training step with respect to the ANN applied to the aluminum plate.

The main idea is to verify if the ANN developed and trained for an isotropic plate is capable of predicting damage also for a composite one. According to the results shown in Figure 16, the ANN produced accurate results. Moreover, the distances between the real damage coordinates and the predicted ones are reported in Table 7. As visible, the distance (error) is lower than 73 mm, and that can be considered to be acceptable, taking into account the previous considerations about the ANN development and the fact that the ANN was trained by using only the aluminum dataset. The error is expected to decrease with an improved training phase. It must also be noticed that the error equal to 73 mm represents a singularity. In fact, according to Table 7, the other errors range between 7.8 and 29.3 mm.

## 6. Conclusions

In this paper, a machine learning approach based on ANN for damage detection and localization was proposed. Specifically, this paper dealt with the development and the assessment of an ANN for the damage detection based on the guided wave propagation method. The ANN was developed and assessed through the Finite Element Method and then used to simulate guided wave propagation in an aluminum plate under different damage configurations. A first step of the research activities herein proposed was addressed to the development of a reliable FE model aimed to calculate the $S_{0}$ and $A_{0}$ dispersion curves. In particular, three FE modeling techniques were investigated: the former based on 2D-Shell elements, the second based on 3D-Shell elements and the latter based on 3D-Solid elements. The reliability of the modeling techniques was assessed against experimental data, as well as semi-analytic ones provided by Dispersion Calculator software. According to the numerical–experimental–analytical comparison of the results, 2D-Shell FE model was chosen for the development of the dataset useful for the ANN, allowing achieving the highest level of accuracy to the computational time ratio. The possibility to use an accurate and faster method to train an ANN is of relevant importance, enabling the training with respect to a wider dataset.

Concerning the development of the ANN, it was trained with respect to an aluminum plate under different damage configurations. Square-shaped damages with a size of 5 and 10 mm were modeled at different points of the plate. The Damage Index dataset was used as the input vector for the ANN, while the coordinates of the modeled damages were the targets. The procedure is based on an in-house code that automatically extracts the $S_{0}$ mode from the baseline and actual states of the structure and calculates the DIs.

The trained ANN showed a good performance in terms of regression (even if a small dataset was used for the training). The ANN was found to be able to detect all damages with an acceptable level of accuracy. Because of the proven ANN reliability, its detection capability was also assessed on a different plate made of CFRP laminate, modeled through FEM. It is important to highlight that data achieved by the simulations involving the CFRP plate were not used for the ANN training phase. Nevertheless, the ANN showed its reliability in damage detection yet again.

## Figures and Tables

**Table 1:** Material properties of Al 6061 plate and PIC255 sensors.

Material Properties	Symbol	Units	Al 6061	PIC255
Mass density	ρ	$\left[{\mathrm{kg}\;m}^{-3}\right]$	2700	7850
Young’s modulus	E	[GPa]	69	76
Shear modulus	G	[GPa]	26	29
Poisson’s ratio	ν	−	0.33	0.32
Dielectric constant	$K_{3}$	−	−	1280
Piezoelectric charge constant	$d_{31}$	$\left[10^{-9}{\;\mathrm{mm}\;V}^{-1}\right]$	−	−180

**Table 2:** Comparison of the relative error for the estimation of the S_0_ mode group velocity for each element type with respect to the experiment—300 kHz carrier.

Data	c_g_ (m/s)	Error (%)
Experimental	5201	−
Semi-Analytical	5268	−1.28
2D-Shell (20 NPW)	4996	3.94
3D-Shell (20 NPW)	5098.9	1.96
3D-Solid (10 NPW)	5117.4	1.60

**Table 3:** Percentage deviation between 20 NPW 2D-Shell FE and experimental group velocity values extracted from the 2nd-order polynomial interpolation.

Frequency (kHz)	$c_{g}$ [m/s] $S_{0}\mathrm{Mode}$			$c_{g}$ [m/s] $A_{0}\mathrm{Mode}$		
	Experimental	20 NPW 2D-Shell	Difference (%)	Experimental	20 NPW 2D-Shell	Difference (%)
50	5315	5427	−2.10	1665	1561	6.25
100	5296	5369	−1.36	2184	2127	2.61
150	5277	5314	−0.69	2526	2550	−0.95
200	5256	5258	−0.02	2748	2888	−5.08
250	5231	5205	0.51	2883	3141	−8.94
300	5201	5154	0.92	2950	3310	−12.2
350	5164	5103	1.19	2990	3110	−4.01
400	5118	5054	1.26	2995	3118	−4.11
450	5061	5006	1.11	2996	3054	−1.94
500	4993	4960	0.67	2958	2920	2.60

**Table 4:** Percentage deviation between 20 NPW 2D-Shell FE and semi-analytical group velocity values extracted from the 2nd-order polynomial interpolation.

Frequency (kHz)	$c_{g}$ [m/s] $S_{0}\mathrm{Mode}$			$c_{g}$ [m/s] $A_{0}\mathrm{Mode}$		
	Semi-Analytical	20 NPW 2D-Shell	Difference (%)	Semi-Analytical	20 NPW 2D-Shell	Difference (%)
50	5352	5427	−1.40	1735	1561	10.03
100	5348	5369	−0.39	2133	2127	0.28
150	5337	5314	0.43	2465	2550	−3.45
200	5320	5258	1.17	2732	2888	−5.71
250	5296	5205	1.72	2932	3141	−7.13
300	5266	5154	2.13	3068	3310	−7.89
350	5229	5103	2.41	3138	3110	0.89
400	5186	5054	2.55	3142	3118	0.76
450	5136	5006	2.53	3081	3054	0.88
500	5080	4960	2.36	2955	2920	1.18

**Table 5:** Analyzed damaged configurations.

Configuration #	Damage Size (mm)	Center Coordinates (mm)
$d_{1}$	5/10	[143.5, 259.5]
$d_{2}$	5/10	[259.5, 204.5]
$d_{3}$	5/10	[232, 143.5]
$d_{4}$	5/10	[204.5, 55]
$d_{5}$	5/10	[143.5, 82.5]
$d_{6}$	5/10	[70.5, 70.5]
$d_{7}$	5/10	[40, 143.5]
$d_{8}$	5/10	[82.5, 204.5]
$d_{9}$	5/10	[143.5, 143.5]
$d_{10}$	10	[60, 120]
$d_{11}$	10	[55, 165]
$d_{12}$	10	[143, 212]
$d_{13}$	10	[168, 222]
$d_{14}$	10	[216, 216]
$d_{15}$	10	[215, 100]
$d_{16}$	10	[190, 180]
$d_{17}$	10	[110, 110]
$d_{18}$	10	[175, 115]
$d_{19}$	10	[120, 168]

**Table 6:** Lamina mechanical properties of CFRP composite.

E_11_ (GPa)	E_22_ (GPa)	E_33_ (GPa)	G_12_ (GPa)	G_13_ (GPa)	G_23_ (GPa)	ν_12_	ν_13_	ν_23_	ρ (kg m^−3^)
105	7.7	7.7	3.6	3.6	2.7	0.36	0.36	0.4	1540

**Table 7:** ANN accuracy for damage prediction in the composite panel.

Configuration #	Real Damage Coordinates (mm)	ANN Predicted Damage Coordinates (mm)	Distance (Error) between Points (mm)
$d_{1}$	[143.5, 259.5]	[96.4, 203.5]	73.1
$d_{5}$	[143.5, 82.5]	[141.9, 90.2]	7.8
$d_{7}$	[40, 143.5]	[27.7, 135.3]	14.8
$d_{16}$	[190, 180]	[219.2, 177.7]	29.3

## Data Availability

Not applicable.
